# An Observational Evaluation of Move-To-Improve, a Classroom-Based Physical Activity Program, New York City Schools, 2010

**DOI:** 10.5888/pcd9.120072

**Published:** 2012-09-13

**Affiliations:** Author Affiliations: Jazmine A. Venturanza, The Fund for Public Health in New York, New York; Rhonda J. Walsh, Cathy A. Nonas, New York City Department of Health and Mental Hygiene, New York, New York.

## Abstract

**Introduction:**

Few children in the United States achieve the recommended 60 minutes of physical activity per day. Identifying successful interventions that increase physical activity for children is critical. This observational study evaluated the effects of Move-To-Improve (MTI), a classroom-based physical education program designed for kindergarten to third-grade teachers in New York City public schools. MTI organizes 3-hour trainings for teachers that demonstrate strategies for integrating activity into daily classroom schedules.

**Methods:**

Randomly sampled elementary schools (N = 39) with classrooms trained in MTI in spring 2010 participated in the evaluation. In each school, we observed 2 classrooms trained in MTI and 2 untrained classrooms in the same school matched by grade level for 1 full school day. We analyzed data from 72 trained and 72 untrained classrooms.

**Results:**

Ninety-nine percent of MTI-trained classroom teachers led their students in physical activity. MTI-trained classrooms spent an average of 9.5 minutes in physical activity per day, compared with 2.4 minutes in untrained classrooms (*P* < .001), an almost fourfold increase in activity. Levels of activity were higher in trained versus untrained classrooms regardless of grade level or class size.

**Conclusion:**

Teachers trained in MTI led their classrooms in significantly more physical activity compared with teachers who were not trained. The MTI program is an effective strategy for increasing physical activity during the school day. A curriculum that empowers classroom teachers to incorporate activity into their regular day is a practical approach to promoting healthier living for children.

## Introduction

The benefits of physical activity among healthy children are well-researched (1-3) and recognized (4-8). Higher levels of activity are correlated with reduced risks for type 2 diabetes, cardiovascular disease, depression, and other diseases (9-11). In addition, physical activity plays an important role in maintaining a healthy weight (12).

Although the Centers for Disease Control and Prevention (CDC) recommends that all children receive at least 60 minutes of daily physical activity, only 49% of boys and 35% of girls in the United States aged 6 to 11 years meet these guidelines (13). Children spend a substantial amount of time in school, yet they have little to no physical activity. Only 4% of elementary schools nationwide provide daily physical education (PE) (14). In 2010, the New York City (NYC) Comptroller's Office conducted an audit of 31 public elementary schools to assess compliance with the New York State Education Department's regulation requiring schools to provide 120 minutes of PE per week to all students (15). Only 2 of the schools met this goal.

Financial constraints and pressure to improve test scores have led schools to reduce PE and school-based physical activity programs (16). However, no clear evidence indicates that academic achievement improves when PE classes are eliminated (17). To the contrary, studies show that spending more time in PE and other school-based activity does not harm academic performance (18,19). Students with increased PE time in schools perform better on standardized tests (19) and can improve on-task behavior during academic instruction (16). In NYC, students with better physical fitness in the 4th and 8th grades were found to have higher test scores (20).

To increase children's physical activity, the NYC departments of Health and Mental Hygiene (DOHMH) and Education (DOE) launched the Move-To-Improve (MTI) program for kindergarten, first-, second-, and third-grade teachers in NYC public elementary schools (21). The goal of the MTI program is to support state PE requirements by enabling classroom teachers to integrate activity into their classrooms through fitness breaks. The MTI program, cowritten by the DOHMH and DOE, integrates core academic requirements into fitness breaks, allowing teachers to increase physical activity for their students while reinforcing grade-level academic concepts. All activities were created for small classroom spaces and do not depend on gyms or multipurpose rooms, which few urban schools have. All fitness breaks count toward the 120 minutes that the state mandates.

The objectives of this evaluation were to determine whether MTI training increases physical activity time for students in classrooms with trained teachers and to examine which classroom characteristics are correlated with use of the curriculum after one 3-hour teacher training.

## Methods

The NYC DOHMH and DOE institutional review boards approved the study protocol. All NYC public elementary schools were invited to apply to MTI. Invitations were included in regular weekly e-mails from the DOE to all elementary school principals and distributed to principals and school administrators. All schools that completed an online grant application were awarded the grant. Participating elementary schools were asked to send a minimum of 6 teachers to one 3-hour MTI workshop held after school or on weekends. At the workshop, an MTI trainer discussed childhood obesity and the importance of physical activity, reviewed the MTI manual, led them through 17 of the 30 MTI fitness breaks, and asked teachers to discuss how they could adapt lessons to their classrooms. Teachers received 1) a manual detailing 30 fitness breaks; 2) equipment kits including polyvinyl spot markers used to help children identify personal space, bean bags, scarves, and 2 CDs; and 3) a professional development stipend of $68.14 based on the United Federation of Teachers (UFT) negotiated training rate. In the 2010-11 school year, the DOHMH trained more than 1,200 teachers from 160 public schools in the MTI program.

To develop the sampling frame, we generated a list of all 147 elementary schools trained in MTI during the spring semester of the previous school year (February to May 2010). Schools exclusively serving special needs students were excluded from this list (n = 4) because their experience with the program is likely to be different from that of traditional schools. Additionally, schools with fewer than 4 MTI-trained staff (n = 37) were excluded because they failed to send at least 6 teachers for training. Finally, schools with more than 19 MTI-trained staff (n = 1) were excluded because such a high level of training does not reflect the experience of most schools. A total of 105 schools remained in the sampling frame. Schools were contacted for participation starting from the top of the randomized list; our goal was to recruit 40 schools. Forty schools with 2 trained and 2 untrained classrooms would ensure adequate power to detect an estimated difference of 5 minutes of activity between trained and untrained classrooms. If a school did not respond to at least 10 attempts to contact them or refused participation, program staff approached the next school on the randomized list. To limit seasonal variation in physical activity, data collection was limited to a 2-month window; observations ended on December 17, 2010.

Once a school agreed to participate, a randomized list of trained teachers from that school was generated by computer. An MTI program representative met with the school principal to review the purpose of the evaluation, the evaluation protocol, and the risks and benefits of participation. The MTI program representative selected the first 2 teachers from the randomized list for observation. Principals then identified 2 untrained classroom teachers in the same school matched by grade level. When a classroom of the same grade was not available, the principal selected 1 from 1 grade higher or lower (n = 9). All 4 classroom observations were scheduled for the same day when none of the participating classrooms had PE or gym class.

Of the 39 schools, 150 classrooms were observed. Five trained teachers and 1 untrained teacher were absent on the day of observation, and no suitable replacement was available. Of the observed classrooms, 1 classroom was excluded from analysis because the amount of activity observed was substantially greater than that of any other classroom, and 5 were excluded because they lacked a matched pair. A total of 72 MTI-trained classrooms and 72 untrained classrooms matched by grade level were included in the final analysis.

After a 2-day training session, data collectors were paired for the first 2 observations, and each independently followed the observation protocol. After the 2 observations, data collectors met with the evaluation team to ask and answer questions and clarify inter-observer disagreement.

Full-day classroom observations took place from mid-October to mid-December 2010. Ten data collectors were trained to conduct the observations in early October. A rotating team of 4 data collectors visited each participating school; 1 data collector was assigned to each classroom, and data collectors were blinded to the training status of their assigned teacher. On arriving at the school, they reported to the main office and then to their assigned classrooms to obtain teacher consent. They informed teachers that they were there to evaluate the MTI program, not to assess the way teachers manage their classrooms. Additionally, data collectors stressed that teachers should not alter their scheduled day. If a teacher refused to participate, data collectors contacted an MTI program staff member, who assigned a replacement teacher for observation from the randomized lists (n = 4). Data collectors remained in the classroom for the full day, during which they recorded 1) all instances of physical activity observed in the classroom; 2) how long those activities lasted; 3) a brief description of the activity; 4) whether teachers encouraged students to be active; 5) whether teachers demonstrated activities to their students (either before or during the activity); 6) when lessons were used; and 7) whether activities reinforced academic concepts. Activities were categorized as high- or low-energy. Activities were considered high-energy if they provided continuous movement (eg, running, jumping, squatting, dancing). Low-energy activities were defined as those with limited movement (eg, stretching, yoga, deep-breathing).

Descriptive statistics were calculated and independent *t* tests performed to evaluate differences in mean minutes of activity observed between trained and untrained classrooms. Analysis of variance and *t* tests were used to assess the differences in activity by grade level and classroom size. Linear regression was conducted to determine whether MTI training affected observed minutes of physical activity after controlling for class size, grade level, and access to PE and recess. Data were analyzed in SPSS for Windows version 18.0 (IBM, Chicago, Illinois).

Teachers trained in MTI led students in 7.1 minutes more physical activity (*P* < .001). With each increase in grade level, activity time decreased by 1.3 minutes (*P* = .005). Class size and access to physical activity did not predict physical activity.

## Results

In classrooms where physical activity took place, students with teachers trained in MTI had significantly more physical activity than students in classrooms without a trained teacher (9.6 minutes vs 4.5 minutes, *P* < .001). These students also experienced more instances of physical activity during the day (3.3 times vs 2.3 times; *P* = .02). Additionally, teachers trained in MTI led their students in significantly more high-energy activities (2.2 activities vs 1.0 activities; *P* < .01) and demonstrated more of these activities for their students (2.7 activities vs 1.7 activities; *P* = .02). Trained and untrained classrooms did not differ by number of activities used to reinforce academic concepts.

Of the 73 elementary schools invited, 16 (22%) did not respond. Of the remaining 57 schools, 3 (5%) were ineligible because of an insufficient number of MTI-trained teachers, and 15 (26%) refused participation. A total of 39 schools completed the evaluation, representing a 68% participation rate. Schools varied by location, availability of physical activity facilities, percentage of students participating in the free lunch program, and prevalence of MTI training (Table 1).

**Table 1:** Characteristics of Schools (N= 39) Participating in Move-To-Improve, New York City, 2010

Characteristic	No. (%)
**Borough**	
Bronx	9 (23.1)
Brooklyn	17 (43.5)
Manhattan	3 (7.7)
Queens	8 (20.5)
Staten Island	2 (5.1)
**Facilities**	
Gym	35 (89.7)
Nearby park	18 (46.2)
Playground	34 (87.2)
Multipurpose room	34 (87.2)
**% Eligible for free lunch^a^**	
<50	4 (11.1)
50–75	8 (22.2)
>75	24 (66.6)
**MTI-trained school staff**	
4 or 5 teachers	10 (25.6)
6–10 teachers	26 (66.6)
11–15 teachers	3 (7.8)
Principal or AP trained	4 (10.3)

Abbreviations: MTI, Move-to-Improve; AP, assistant principal.

^ a ^Refers to students who are eligible for free or reduced-cost lunch through the National School Lunch Program.

Classrooms varied by grade level, size, classroom type, and access to physical activity (Table 2). Overall, MTI-trained classrooms participated in significantly more physical activity than untrained classrooms (Table 3). In 71 of 72 MTI-trained classrooms, teachers led their students in physical activity. Trained classroom teachers led an average of 3 activities per day; each activity lasted approximately 3 minutes. Students with trained teachers participated in a total of 9.5 minutes of physical activity. In contrast, 38 of the 72 classrooms with untrained teachers were led in physical activity. Students with untrained teachers averaged 1 activity per day and were active for an average of 2.4 minutes.

**Table 2:** Classroom Characteristics, Move-To-Improve, New York City Schools, 2010

Characteristics	All Classrooms, n (%) (N = 144)	Trained Classrooms, n (%) (n = 72)	Untrained Classrooms, n (%) (n = 72)
**Grade level**			
Kindergarten	33 (22.9)	16 (22.2)	17 (23.6)
First	41 (28.5)	24 (33.3)	17 (23.6)
Second	38 (26.4)	17 (23.6)	21 (29.2)
Third	32 (22.2)	15 (20.8)	17 (23.6)
**Classroom size**			
<20 students	34 (23.6)	15 (20.8)	19 (26.4)
20–25 students	89 (61.8)	46 (63.9)	43 (59.7)
26–30 students	21 (14.6)	11 (15.3)	10 (13.9)
**Classroom type**			
General education	125 (86.8)	59 (81.9)	66 (91.7)
Collaborative team teaching^a^	17 (11.8)	11 (15.3)	6 (8.3)
Special education	1 (0.7)	1 (1.4)	0
English as a second language	1 (0.7)	1 (1.4)	0
**Access to physical activity**			
Classrooms with physical education class	132 (91.7)	65 (49.2)	67 (50.8)
Classrooms with no physical education class	12 (8.3)	7 (9.7)	5 (6.9)
Classrooms with recess	128 (88.2)	65 (50.4)	63 (9.6)
Classrooms with no recess	16 (11.1)	8 (50.0)	8 (50.0)

^a^ Collaborative team teaching classrooms integrate special needs students into general education classrooms, often with 2 or more teachers present to address the needs of both populations.

**Table 3:** Classroom Activity by Grade, Size, and Access to Physical Activity, Move-to-Improve, New York City Schools, 2010

Percentage of Classrooms Where Activity Was Observed	All Classrooms (N = 144)	Trained Classrooms (n = 72)	Untrained Classrooms (n = 72)	*P* Value

	Mean (SD)			
**Classrooms observed**	109 (75.7)	71 (98.6)	38 (52.7)	<.001
**Activities observed**	2.2 (2.3)	3.3 (2.2)	1.2 (1.8)	<.001
**Length of total activity time, min **	5.9 (6.7)	9.5 (7.1)	2.4 (3.8)	<.001
**Grade**				
Kindergarten	8.8 (7.7)	14.1 (6.6)	3.8 (4.9)	<.001
First	6.3 (6.4)	9.0 (6.4)	2.3 (3.7)	<.001
Second	4.6 (6.3)	8.2 (7.8)	1.7 (2.2)	.001
Third	4.2 (5.9)	6.8 (6.5)	1.9 (4.2)	.016
**Classroom size**				
<20 students	7.2 (7.2)	12.3 (7.0)	3.1 (4.2)	<.001
20–25 students	6.1 (7.0)	9.5 (7.6)	2.5 (3.9)	<.001
26–30 students	3.2 (3.4)	5.5 (2.9)	0.5 (1.1)	<.001
**Access to physical activity, min**				
Classrooms with PE class (n = 132)	5.9 (6.7)	9.5 (7.1)	2.4 (3.9)	<.001
Classrooms without PE class (n = 12)	6.5 (7.5)	9.6 (8.6)	2.1 (2.0)	.05
Classrooms with recess (n = 128)	5.8 (6.5)	9.1 (6.9)	2.5 (4.0)	<.001
Classrooms without recess (n = 16)	7.0 (8.6)	12.6 (8.9)	1.4 (2.2)	.004

Abbreviation: PE, physical education.

Students in MTI-trained classrooms had significantly more physical activity across grade level, classroom size, and access to physical activity. Overall, in both trained and untrained classrooms, physical activity declined as grade level increased (*P* = .02, analysis of variance). Additionally, physical activity was significantly lower in classrooms with 26 to 30 students than in classrooms with 20 to 25 students and classrooms with fewer than 20 students (*P* < .01, *t *test). Finally, classrooms in schools with MTI-trained principals or assistant principals did not provide significantly more physical activity than classrooms in schools without a trained administrator.

## Discussion

Move-To-Improve (MTI) empowers teachers to increase physical activity in small classroom spaces. As grade level and class size increased, time spent in physical activity declined. In multivariate analysis, grade level was a significant predictor of activity, whereas class size was not. Given that academic pressures are stronger in third-grade classrooms than in kindergarten, teachers in older grades might feel that physical activity in the classroom could detract from academic instruction. Nevertheless, although these data indicate that the effect of MTI is greatest in younger classrooms, they demonstrate that the program can be effective in older grades as well. Additionally, these data suggest that MTI training is effective even for teachers with many students and crowded classrooms.

A number of classrooms in our sample lacked any systematic access to physical activity. Sixteen classrooms had no scheduled recess, and 12 had no scheduled PE. In these classrooms, as in others, MTI training significantly increased observed classroom-based activity. Training classroom teachers may be important to ensure that children receive some opportunity for movement during the school day.

On average, teachers trained in MTI implemented 3 minutes of physical activity several times during the school day. Only 2 studies have examined the effect of very brief bouts of activity on clinical outcomes in children. One compared the effects of moderate-to-vigorous activity lasting less than 5 minutes with bouts lasting more than 5 minutes in 2,754 children aged 6 through 19 and found no difference in cardiometabolic risk factors between children who gained most of their activity through 1 form over the other (22). The other examined the effect of bout length on overweight in 2,498 children aged 8 through 17 and determined that physical activity that took place in bouts lasting longer than 5 minutes conferred some benefits in overweight status over sporadic activity lasting less than 5 minutes (23). To the best of our knowledge, no one has studied the effects of these bouts on children's physical activity behaviors as they age. The state mandates for PE do not establish minimum lengths of activity bouts; instead, they only specify the total number of minutes per week required. Likewise, both CDC and the Institute of Medicine recommend that children obtain 60 minutes of activity per day but make no requirements for bout length. The primary goal of the MTI program is to support elementary schools in their efforts to meet the state's mandate of 120 minutes of PE per week, and this evaluation demonstrates that MTI can be an effective strategy to increase compliance with these regulations. Additional program efforts will encourage teachers to implement MTI activities for longer bouts at a time.

Given the size of many urban school districts, interventions that require substantial investments in individual schools are not cost-effective. Programs that require ongoing technical assistance, refresher trainings, and continual support cannot be sustained in a school system as large as the NYC DOE. With nearly 800 elementary schools and an estimated 14,000 kindergarten through third-grade teachers serving more than 478,000 students in the NYC public school system, the MTI program represents a model intervention that can be successfully implemented, sustained, and adopted in large urban districts. Indeed, from its inception in fall 2009 through spring 2012, the MTI program has trained more than 4,300 teachers from approximately 500 elementary schools in the city's 5 boroughs.

This study had several limitations. First, the only schools eligible for participation in this evaluation were those that had participated in MTI. Because these schools elected to participate in a physical activity training program, they may be more likely to implement the program than the overall population of NYC schools. Second, because we compared trained and untrained teachers from within the same school, untrained teachers may have learned about MTI from their trained counterparts and began implementing activity on their own. However, this would have decreased our likelihood of finding a difference in physical activity time. Third, although trained teachers were randomly selected, untrained teachers were not. These teachers might not have been representative of all untrained teachers. Fourth, observed classroom teachers may have felt compelled to demonstrate more physical activity than they would usually provide during a school day. Although this pressure was presumably similar for trained and untrained teachers, trained teachers may have been more capable of leading fitness activities because of their MTI training. Fifth, observations were completed for only 1 school day when no PE class was scheduled. These data, then, cannot be extrapolated to determine what teachers would do on a day with a scheduled PE period. Sixth, 26% of the schools approached to participate in this study refused. Thus, schools that agreed to participate may have been a biased sample that was more likely to use fitness breaks compared with the overall NYC public school population. Finally, because data collectors were recording observed activity time, slight measurement errors may exist in the amount of time observed. However, the amount of time separating levels of physical activity in trained and untrained classrooms was so significant that measurement errors would not erase these differences.

Teachers were trained in MTI from February through May 2010 and were observed 6 months later, from October to December 2010. Also, because of issues with the MTI program's equipment vendor, most teachers trained in MTI in spring 2010 had not received their MTI equipment by the end of the evaluation. Despite these circumstances, 99% of trained teachers successfully incorporated MTI lessons into their classrooms the following school year, suggesting that MTI training has long-lasting effects.

Despite these limitations, to our knowledge, this is the largest observational evaluation of its kind. It demonstrates that MTI training increases physical activity time for students in the classroom setting. This intervention, requiring only a 1-time training with no follow-up, is feasible for large, urban school districts where time-intensive interventions are financially impossible. Giving classroom teachers the skills to implement PE is an effective approach to increasing physical activity for elementary school students.
